# Supplementary material for: Intelligent Poly(N-Isopropylmethacrylamide) Hydrogels: Synthesis, Structure Characterization, Stimuli-Responsive Swelling Properties, and Their Radiation Decomposition
Source: Polymers (Basel). 2020 May 13;12(5):1112. doi: 10.3390/polym12051112 (PMC7285319; doi:10.3390/polym12051112)
Supplement: Supplementary file 1 [file polymers-12-01112-s001.pdf]

Supplementary Material: Intelligent Poly(*N*-Isopropylmethacrylamide) Hydrogels: Synthesis, Structure Characterization, Swelling Properties, and Their Radiation Decomposition

Snežana Ilić-Stojanović [1,*], Maja Urošević [1], Ljubiša Nikolić [1], Djordje Petrović [2], Jelena Stanojević [1] Stevo Najman [3], Vesna Nikolić [1]

[1] University of Niš, Faculty of Technology, Bulevar oslobodjenja 124, 16000 Leskovac, Republic of Serbia; snezanai@tf.ni.ac.rs (S.I.-S.), maja@tf.ni.ac.rs (M.U.), nljubisa@tf.ni.ac.rs (L.N.), jstanojevic@tf.ni.ac.rs (J.S.), nikolicvesna@tf.ni.ac.rs (V.N.)

[2] Vinča Institute of Nuclear Sciences, Department of Radioisotopes, University of Belgrade, Mike Petrovića Alasa 12-14, 11351 Vinča, Belgrade, Republic of Serbia; djpetrovic@vin.bg.ac.rs (D.P.)

[3] University of Niš, Faculty of Medicine, Niš, Blvd. Dr Zorana Djindjica 81, 18108 Niš, Republic of Serbia; stevo.najman@medfak.ni.ac.rs (S.N.)

* Correspondence: ilic.s.snezana@gmail.com; (S.I.-S.)

Table S1. Peak surface values for series of standard *N*iPMAm solutions with HPLC chromatogram at R_t=2.506 min.

c_{NiPMAm}. mg/cm^3	1.028	0.771	0.514	0.257	0.1028	0.0514	0.0257	0.01028	0.00514
A_{212nm}. mAU·s	14198.5	13285.1	12023.7	9720.6	5877.4	5877.4	1656.4	698.8	396.3

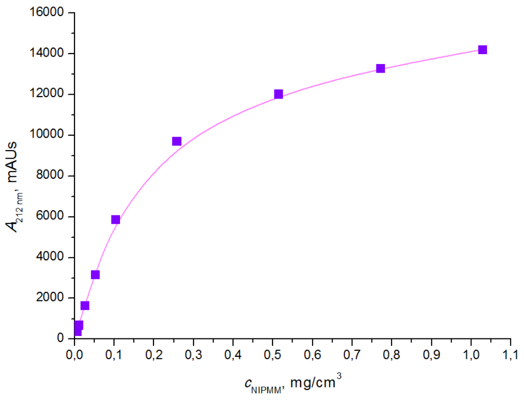

Figure S1. Calibration curve: dependence of peak area from HPLC chromatogram versus *N*-isopropylmethacrylamide (*N*iPMAm) concentration; Rt = 2.506 min.

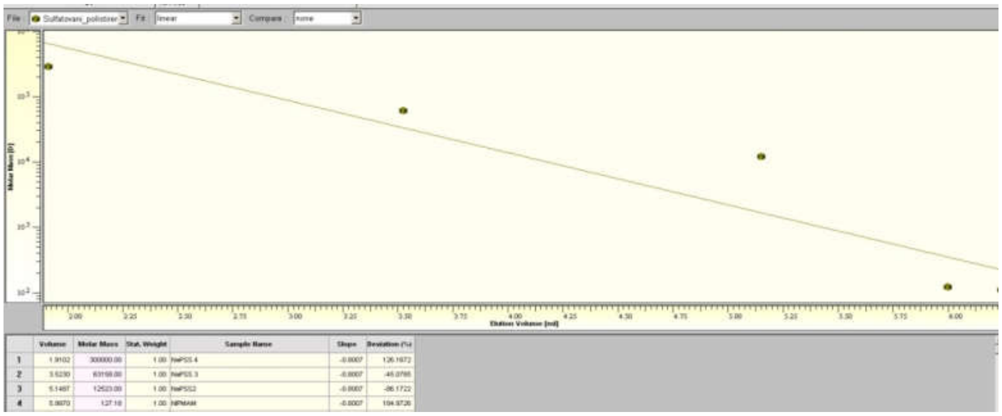

Figure S2. Calibration curve for GPC obtained by standards solutions of *N*iPMAm and sulfonated poly(styrene) with narrow molecular weight distributions

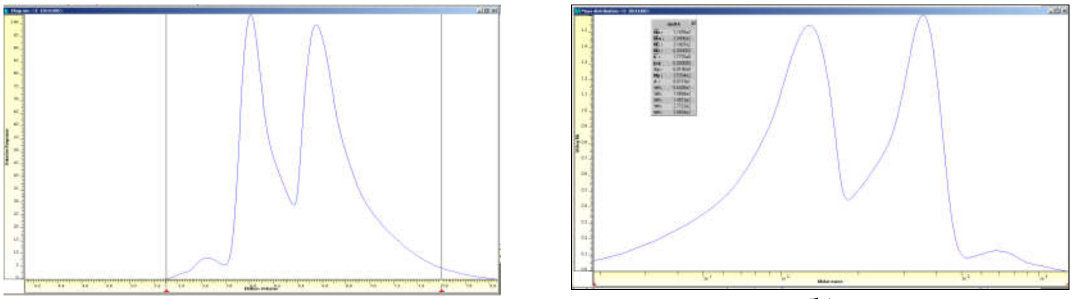

(**a**) (**b**)

Figure S3. (a) Elugram and (b) differential molar mass distribution of liquid material after poly(*N*-isopropylmethacrylamide) (p(*N*iPMAm)) gamma irradiation, for fractions 5.30-7.60 min.

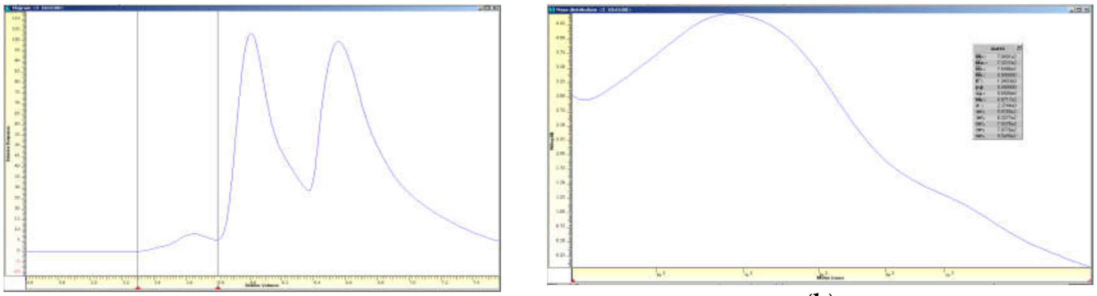

(**a**) (**b**)

Figure S4. (**a**) Elugram and (**b**) differential molar mass distribution of liquid material after p(*N*iPMAm) gamma irradiation, for fractions 5.30-5.80 min.

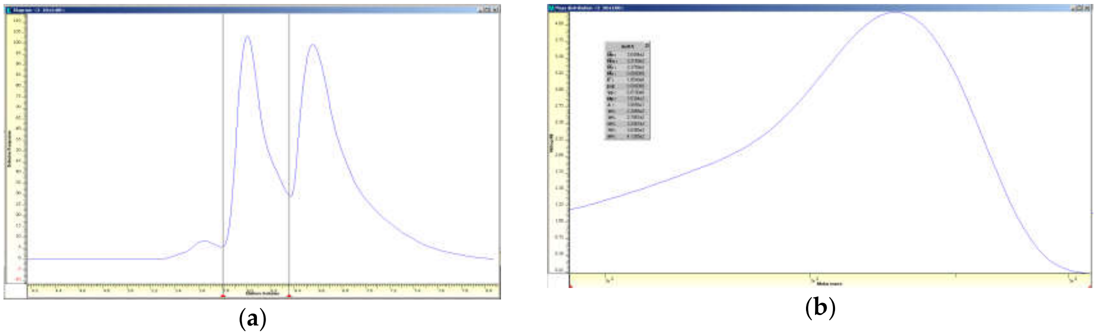

(**a**) (**b**)

Figure S5. (**a**) Elugram and (**b**) differential molar mass distribution of liquid material after p(*N*iPMAm) gamma irradiation, for fractions 5.80-6.36 min.

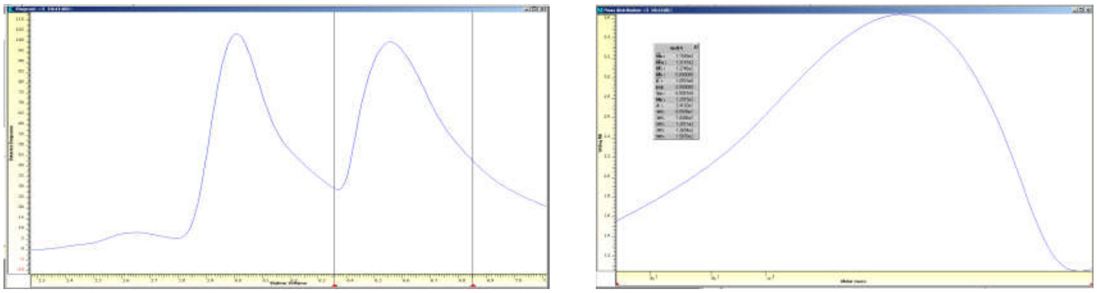

(**a**) (**b**)

Figure S6. (**a**) Elugram and (**b**) differential molar mass distribution of liquid material after p(*N*iPMAm) gamma irradiation, for fractions 6.36-6.85 min.

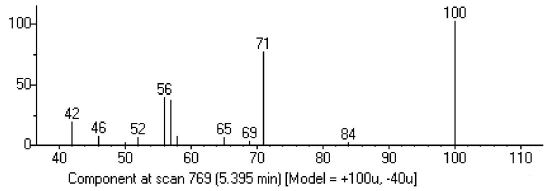

(a)

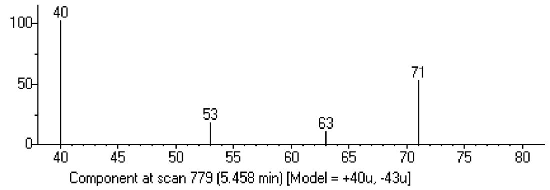

(b)

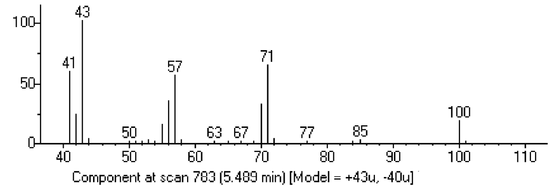

(c)

Figure S7. The deconvoluted mass spectra of the co-eluting components under the peak 1 at 60 °C oven temperature designated by the order of their elution from the HP-5ms column ($t_{ret.}$): **(a)** 5.395 min, **(b)** 5.458 min, **(c)** 5.489 min. The proposed structures are that of heptane **(c)**, heptane isomer **(a)** and related compound **(b)**.

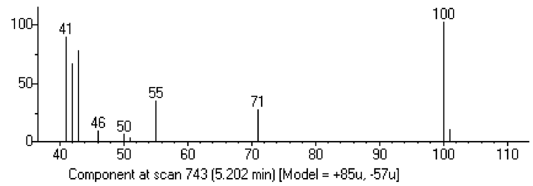

(a)

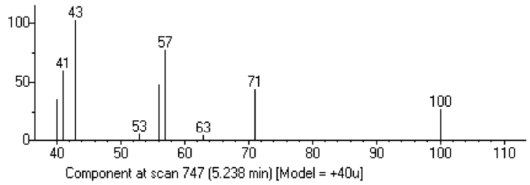

(b)

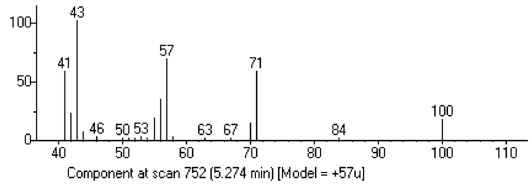

(c)

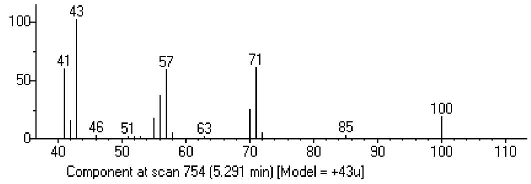

(d)

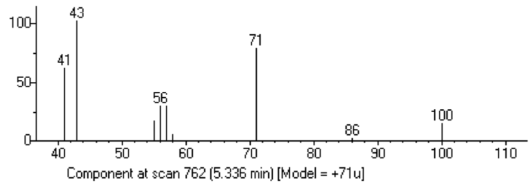

(e)

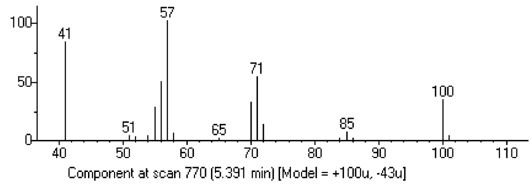

(f)

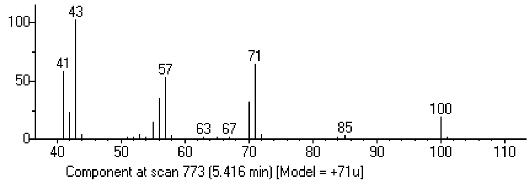

(g)

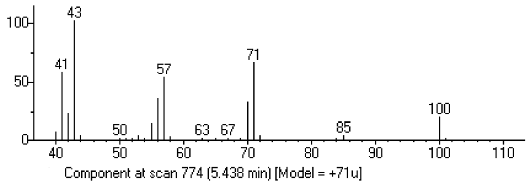

(h)

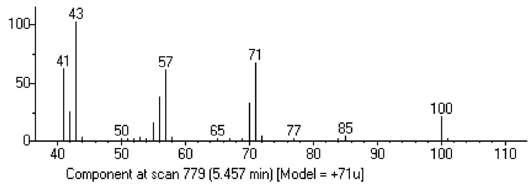

(i)

Figure S8. The deconvoluted mass spectra of the co-eluting components under the peak 1 at 90 °C oven temperature designated by the order of their elution from the HP-5ms column ($t_{ret.}$): **(a)** 5.202 min, **(b)** 5.238 min, **(c)** 5.274 min, **(d)** 5.291, **(e)** 5.336 min, **(f)** 5.391 min, **(g)** 5.438 min, **(i)** 5.457 min. The proposed structures are that of heptane **(i)**, heptane isomers **(b-d, f-h)** and related compounds **(a, e)**.

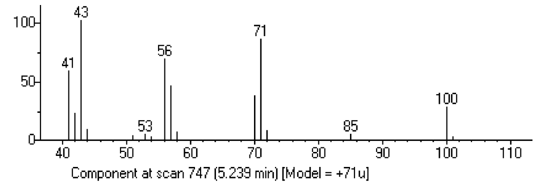

(a)

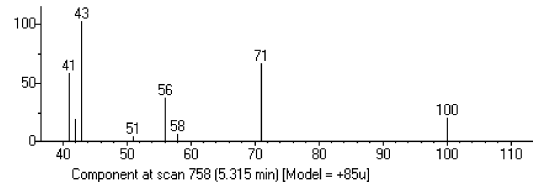

(b)

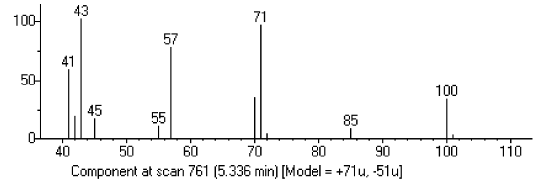

(c)

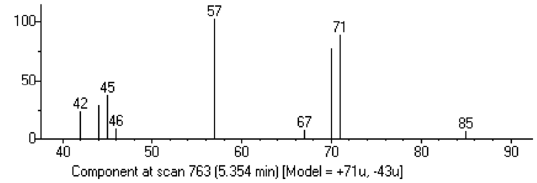

(d)

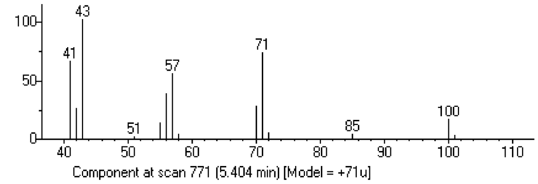

(e)

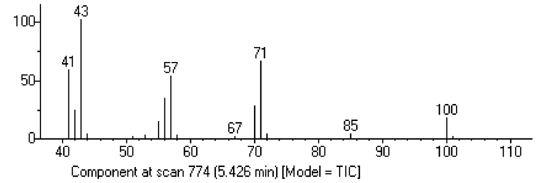

(f)

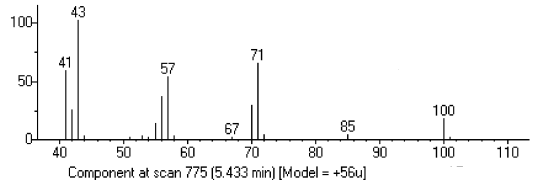

(g)

Figure S9. The deconvoluted mass spectra of the co-eluting components under the peak 1 at 120 °C oven temperature designated by the order of their elution from the HP-5ms column ($t_{ret.}$): **(a)** 5.239 min, **(b)** 5.315 min, **(c)** 5.336 min, **(d)** 5.354 min, **(e)** 5.404 min, **(f)** 5.426 min, **(g)** 5.433 min. The proposed structures are that of heptane **(g)**, heptane isomers **(c, e, f)** and related compounds **(a, b, d)**.

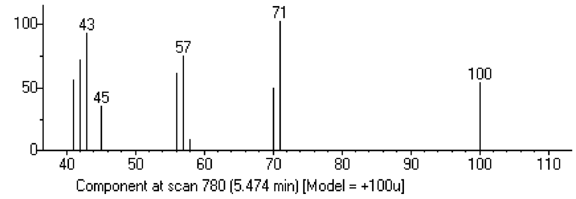

Figure S10. The deconvoluted mass spectrum of the component under the peak 1 at 150 °C oven temperature, eluted at t_{ret}=5.474 min. The proposed structure is that of a heptane.

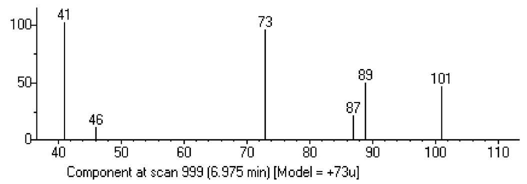

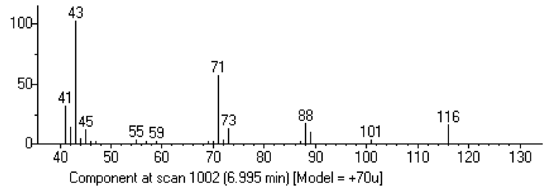

(a) **(b)**

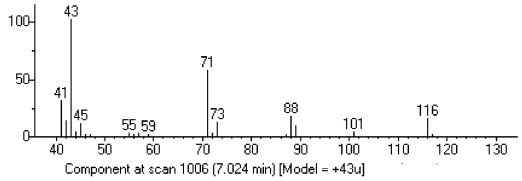

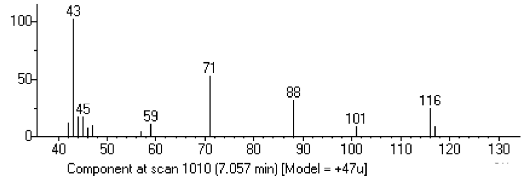

(c) **(d)**

Figure S11. The deconvoluted mass spectra of the co-eluting components under the peak 2 at 60 °C oven temperature designated by the order of their elution from the HP-5ms column ($t_{ret.}$): **(a)** 6.975 min, **(b)** 6.995 min, **(c)** 7.024 min, **(d)** 7.057min. The proposed structures are that of ethyl isobutyrate **(d)**, its isomers **(b, c)** and related compound **(a)**.

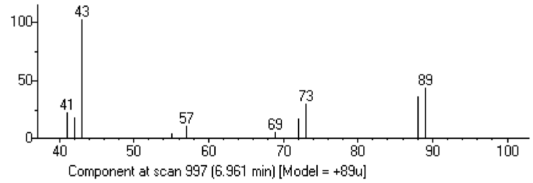

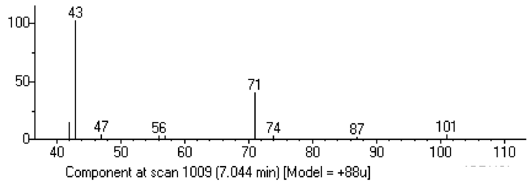

(a) **(b)**

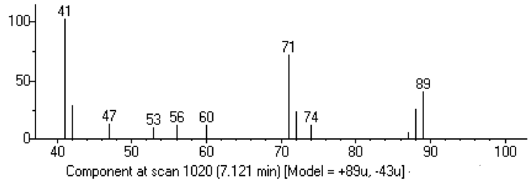

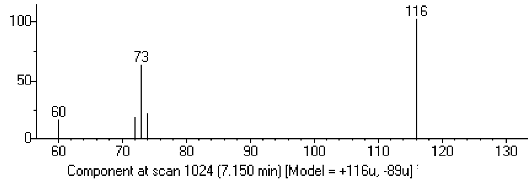

(c) **(d)**

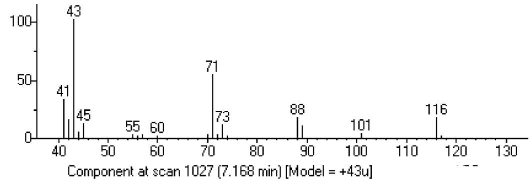

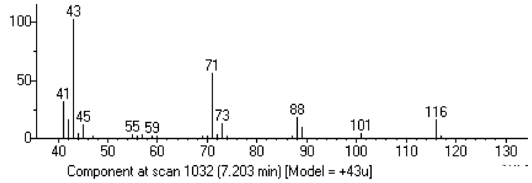

(e) **(f)**

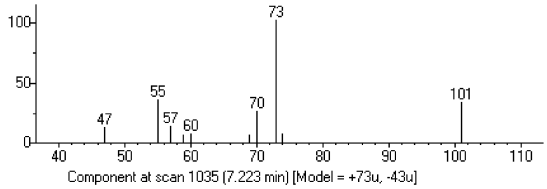

(g)

Figure S12. The deconvoluted mass spectra of the co-eluting components under the peak 2 at 90 °C oven temperature designated by the order of their elution from the HP-5ms column ($t_{ret.}$): **(a)** 6.961 min, **(b)** 7.044 min, **(c)** 7.121 min, **(d)** 7.150min, **(e)** 7.168 min, **(f)** 7.203 min, **(g)** 7.223 min. The proposed structures are that of ethyl isobutyrate **(f)**, its isomer **(e)** and related compounds **(a-d, g).**

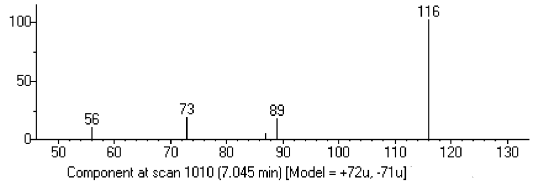

(a)

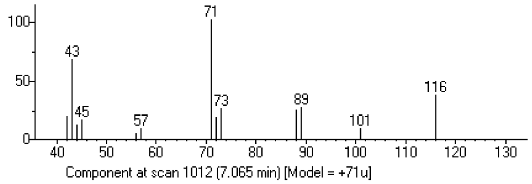

(b)

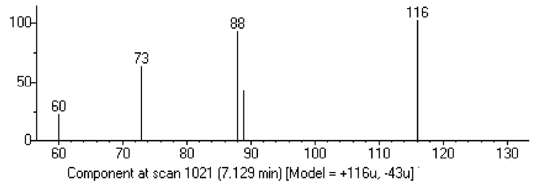

(c)

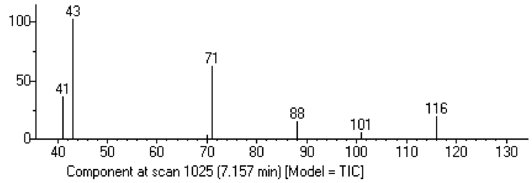

(d)

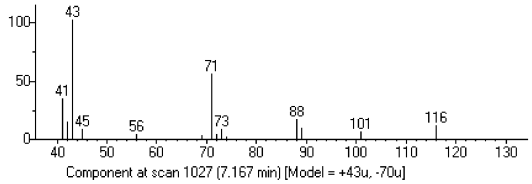

(e)

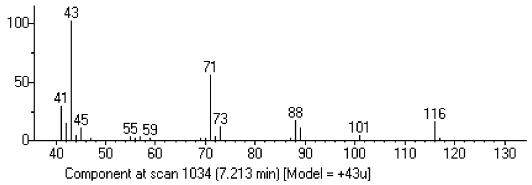

(f)

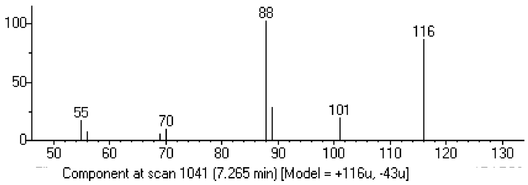

(g)

Figure S13. The deconvoluted mass spectra of the co-eluting components under the peak 2 at 120 °C oven temperature designated by the order of their elution from the HP-5ms column ($t_{ret.}$): **(a)** 7.045 min, **(b)** 7.065 min, **(c)** 7.129 min, **(d)** 7.157min, **(e)** 7.167 min, **(f)** 7.213 min, **(g)** 7.265 min. The proposed structures are that of ethyl isobutyrate **(f)**, its isomers **(d, e)** and related compounds **(a-c, g).**

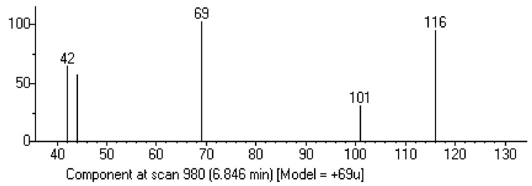

(a)

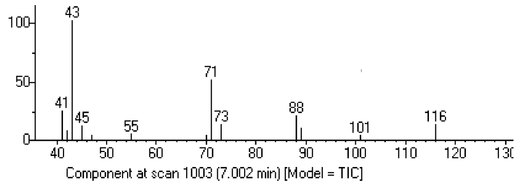

(b)

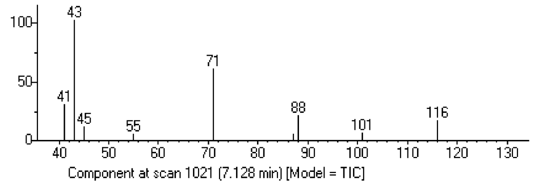

(c)

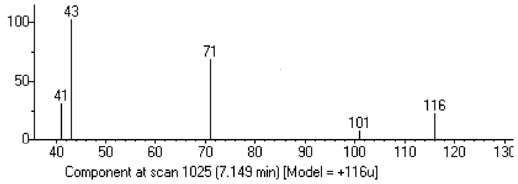

(d)

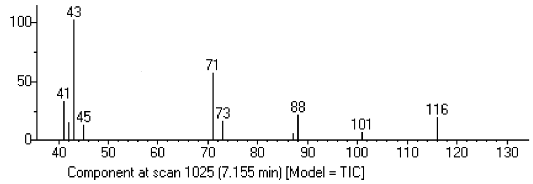

(e)

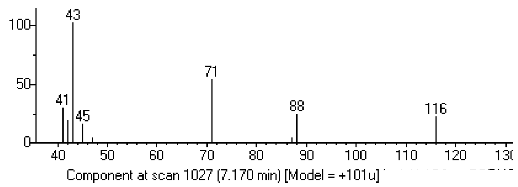

(f)

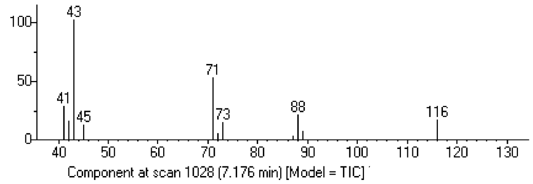

(g)

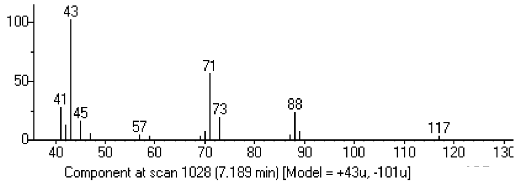

(h)

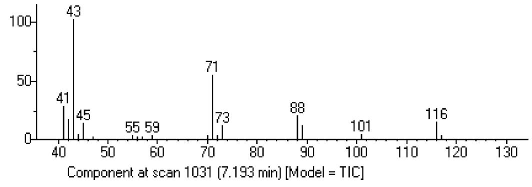

(i)

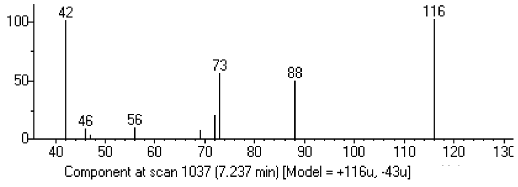

(j)

Figure S14. The deconvoluted mass spectra of the co-eluting components under the peak 2 at 150 °C oven temperature designated by the order of their elution from the HP-5ms column ($t_{ret.}$): **(a)** 6.846 min, **(b)** 7.002 min, **(c)** 7.128 min, **(d)** 7.149min, **(e)** 7.155 min, **(f)** 7.170 min, **(g)** 7.176 min, **(h)** 7.189 min, **(i)** 7.193 min, **(j)** 7.237 min. The proposed structures are that of ethyl isobutyrate **(i)**, its isomers **(b, c, e)** and related compounds **(a, d, f, g, j).**
